# Enlarged Perivascular Spaces Are Negatively Associated With Montreal Cognitive Assessment Scores in Older Adults

**DOI:** 10.3389/fneur.2022.888511

**Published:** 2022-07-01

**Authors:** Timothy J. Libecap, Valentinos Zachariou, Christopher E. Bauer, Donna M. Wilcock, Gregory A. Jicha, Flavius D. Raslau, Brian T. Gold

**Affiliations:** ^1^Department of Neuroscience, College of Medicine, University of Kentucky, Lexington, KY, United States; ^2^Department of Physiology, College of Medicine, University of Kentucky, Lexington, KY, United States; ^3^Sanders-Brown Center on Aging, College of Medicine, University of Kentucky, Lexington, KY, United States; ^4^Department of Neurology, College of Medicine, University of Kentucky, Lexington, KY, United States; ^5^Department of Radiology, College of Medicine, University of Kentucky, Lexington, KY, United States; ^6^Magnetic Resonance Imaging and Spectroscopy Center, College of Medicine, University of Kentucky, Lexington, KY, United States

**Keywords:** enlarged perivascular spaces—ePVS, cerebral small vessel disease, Montréal Cognitive Assessment—MoCA, neuroimaging biomarkers, white matter hyperintensities—WMH

## Abstract

Emerging evidence suggests that enlarged perivascular spaces (ePVS) may be a clinically significant neuroimaging marker of global cognitive function related to cerebral small vessel disease (cSVD). We tested this possibility by assessing the relationship between ePVS and both a standardized measure of global cognitive function, the Montreal Cognitive Assessment (MoCA), and an established marker of cSVD, white matter hyperintensity volume (WMH) volume. One hundred and eleven community-dwelling older adults (56–86) underwent neuroimaging and MoCA testing. Quantification of region-specific ePVS burden was performed using a previously validated visual rating method and WMH volumes were computed using the standard ADNI pipeline. Separate linear regression models were run with ePVS as a predictor of MoCA scores and whole brain WMH volume. Results indicated a negative association between MoCA scores and both total ePVS counts (*P* ≤ 0.001) and centrum semiovale ePVS counts (*P* ≤ 0.001), after controlling for other relevant cSVD variables. Further, WMH volumes were positively associated with total ePVS (*P* = 0.010), basal ganglia ePVS (*P* ≤ 0.001), and centrum semiovale ePVS (*P* = 0.027). Our results suggest that ePVS burden, particularly in the centrum semiovale, may be a clinically significant neuroimaging marker of global cognitive dysfunction related to cSVD.

## Introduction

Cerebral small vessel disease (cSVD) is a major contributor to cognitive impairment in older adults (1–4). cSVD is often characterized by the presence of lacunes, microbleeds, and white matter hyperintensities (WMHs) on magnetic resonance imaging (5–7). Accumulating evidence suggests that enlarged perivascular spaces (ePVS), may also be a neuroimaging marker of cSVD (5, 8–13). ePVS refer to larger than typical fluid-filled spaces along perforating arteries between the astrocytic endfeet of the blood brain barrier and endothelial cells of the cerebrovascular lumen surrounding small arteries (8, 14, 15). The etiology of ePVS remains unknown but impaired clearance of waste through the brain's glymphatic system and blood-brain-barrier damage may represent contributing factors (16–19).

Enlarged perivascular spaces were initially considered benign radiological findings (1). More recent cross-sectional evidence demonstrates an increase in ePVS with age and vascular risk factors, including WMH volume, suggesting ePVS may be a marker of cSVD (18, 20–25). Accumulating evidence also suggests a negative association between ePVS and cognitive processes including executive function, processing speed, semantic memory, and visuospatial ability (26–32).

Nevertheless, the clinical significance of ePVS is not clear due to mixed findings associating ePVS with the the Mini-Mental State Exam (MMSE), a standardized measure of global cognitive function. Some previous studies reported a negative relationship between ePVS burden and MMSE scores (33–37), while others reported no relationship (27, 38–41).

Furthermore, the relationship between ePVS burden and scores on the Montreal Cognitive Assessment (MoCA), a widely used measure of global cognitive function and clinical diagnostic status (42–44), remains to be described. The MoCA was developed as an alternative measure of global cognitive function and clinical diagnosis to the MMSE. Multiple studies have demonstrated that the MoCA is a more sensitive measure of early cognitive dysfunction than the MMSE (43–46). Given that ePVS are thought to represent a potential marker of early cognitive dysfunction it is important to assess their association with MoCA scores.

Here we addressed this issue using a previously validated, visual rating method for quantification of region-specific ePVS burden (47, 48). In addition, we investigated the relationship between ePVS burden and WMH volume, a well-established neuroimaging marker of cSVD (5, 49, 50). Our hypothesis was that ePVS would be negatively associated with MoCA scores, and positively associated with WMH volume, which would support the hypothesis that ePVS are clinically significant and may be an early marker of vascular cognitive dysfunction.

## Materials and Methods

### Participants

One hundred and eleven community-dwelling older adults were initially recruited for the experiment (64 women, age range 56–86). All participants provided informed consent under a protocol approved by the Institutional Review Board of the University of Kentucky. Participants were recruited from an existing longitudinal cohort at the Sanders-Brown Center on Aging (SBCoA) and the Lexington, KY community. Twenty-eight participants were also co-enrolled in the MarkVCID consortium study (51). Participants completed the Montreal Cognitive Assessment (MoCA) (42) within 6 months of their scan date. Participant MoCA scores ranged from 18 to 30. A total of 72 participants had MoCA scores within the cognitively normal range (26–30) while a total of 39 participants had scores within the mild cognitive impairment (MCI) range (18–25).

Exclusion criteria were significant head injury (defined as loss of consciousness for more than 5 min), stroke, neurological disorders (e.g., epilepsy, Alzheimer's disease) or major psychiatric disorders (e.g., schizophrenia, active clinical depression), claustrophobia, pacemakers, the presence of metal fragments or implants that are incompatible with MRI, or significant brain abnormalities detected during imaging. A neuroradiologist (FDR) evaluated the T1W and FLAIR images for evidence of stroke or other clinically relevant abnormalities. This resulted in exclusion of two participants due to evidence of previous stroke (one participant) and hydrocephalus (one participant), not known at the time of enrollment. Three additional participants were excluded due to MoCA scores suggestive of dementia (MoCA <18), an exclusionary criterion for this study. Finally, one participant was excluded on the basis of being a statistical outlier in WMH volume. Detailed characteristics of the final group of 105 participants involved in data analyses are shown in Table 1.

**Table 1:** Group demographics and mean MoCA scores.

***N***	**105**
Age (years)	69.69 ± 6.31
Sex (F:M)	62:43
Education	15.75 ± 2.75
MoCA	26.10 ± 2.85

*The table lists the mean (±sd) for age, years of education, and MoCA score and the female to male ratio*.

*MoCA, Montreal Cognitive Assessment*.

### Magnetic Resonance Imaging Protocol

Participants were scanned in a Siemens 3T Prisma scanner (software version E11C), using a 64-channel head coil, at the University of Kentucky's Magnetic Resonance Imaging and Spectroscopy Center (MRISC). Prior to scanning, all participants were screened to ensure magnetic safety for scanning. The following scans were acquired: (1) a 3D multi-echo, T1-weighted magnetization prepared rapid gradient echo (T1) scan, (2) a 3D fluid-attenuated inversion recovery (FLAIR) scan, (3) a 3D, multi-echo gradient-recalled echo scan used for quantitative susceptibility mapping (QSM). Several other sequences were collected during the scanning session related to other scientific questions and are not discussed further here.

The T1 sequence covered the entire brain [1 mm isotropic voxels, 256 × 256 × 176 mm acquisition matrix, parallel imaging (GRAPPA) acceleration = 2, repetition time (TR) = 2,530 ms, inversion time = 1,100 ms, flip angle (FA) = 7°, scan duration = 5.88 min] and had four echoes [first echo time (TE1) = 1.69 ms, echo spacing (ΔTE = 1.86 ms)]. The 3D FLAIR sequence covered the entire brain (1 mm isotropic voxels, 256 × 256 × 176 acquisition matrix, TR = 5,000 ms, TE = 388 ms, inversion time = 1,800 ms, scan duration = 6.45 min). A high-resolution, flow compensated, multi-echo, 3D spoiled GRE sequence with eight echoes (TR/TE1/ΔTE/FA = 24ms/2.98ms/2.53ms/15°) was acquired and used to create QSM images as described elsewhere (52). The entire brain was covered [acquisition matrix = 224 × 224 × 144, parallel imaging (GRAPPA) acceleration = 2, 1.2 mm isotropic voxels and scan duration = 6.18 min].

### ePVS Counting

**Figure 1:**
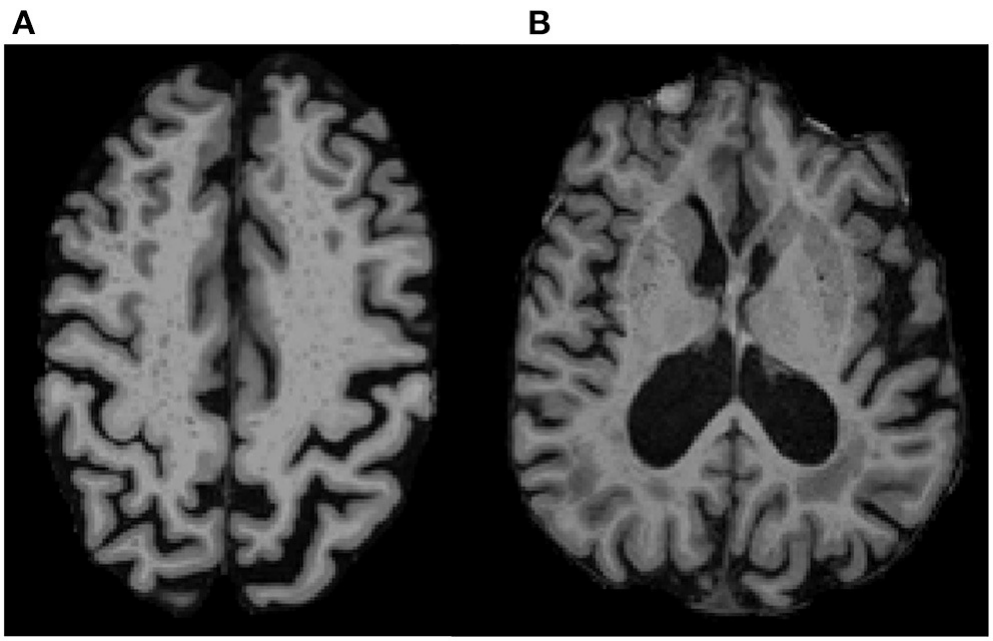
ePVS Regions of Interest on T1 MPRAGE. Examples show ePVS burden in **(A)** the centrum semiovale (white matter; 10 mm above the lateral ventricles) and in **(B)** the basal ganglia (gray matter in the putamen and head of caudate in the plane of the columns of the fornix). A high intra-rater reliability was achieved for ePVS (ICC = 0.9) on a subset of 20 randomly selected participants.

We used a previously validated, visual rating method for quantification of region-specific ePVS burden developed via collaboration between multiple consortia and intended to standardize ePVS assessment across the field of cSVD research (47). The method involves manually counting ePVS on T1 images, with additional reference to T2 FLAIR images. Counts were performed in each hemisphere in a single, axial slice of T1 images, within four regions of interest that are known to have the greatest burden of ePVS (11, 14, 27, 33, 47, 53): the centrum semiovale, 1 cm above the lateral ventricles (Figure 1A); the basal ganglia, in the plane of the columns of the fornix (Figure 1B); the midbrain, at the level of the cerebral peduncles; the hippocampus, at the level of the midbrain.

Previous work by multiple consortia such as STandards for ReportIng Vascular changes on nEuroimaging (STRIVE) and Uniform Neuro-Imaging of Virchow-Robin Spaces Enlargement (UNIVRSE) have demonstrated the reliability of counting ePVS on T1, with very high correlation to counts on T2 images (5, 27, 48) as well as a high correlation between single-slice and multi-slice counts (47, 48, 54–57). A total ePVS score was created for each participant by combining counts across the four ROIs. All counts were conducted by the lead author (TJL) blinded to participant demographics and under the supervision of an experienced neuroradiologist (FDR), who clarified unclear imaging.

In accordance with STRIVE and UNIVRSE consensus guidelines (5, 8, 48, 58), ePVS were identified using T1, FLAIR and susceptibility weighted images. ePVS were identified as hypointense and less than 3 mm in diameter to differentiate them from lacunes, which tend to be larger (5, 59, 60). ePVS were further differentiated from lacunes based on their lack of hyperintensity on FLAIR (5, 48). ePVS were differentiated from cerebral microbleeds (CMBs) by their absence of prominent associated blooming artifact on QSM. We used QSM for differentiation of ePVS and CMBs due to evidence that QSM images outperform traditional single-echo susceptibility weighted images in this regard (61–66). Intra-rater reliability for ePVS was assessed on a subset of 20 randomly selected participants, using intra-class correlation (ICC).

### White Matter Hyperintensity Quantification

Whole brain white matter hyperintensity (WMH) volumes were computed using the ADNI pipeline, specifically the UCD WMH segmentation toolkit (Version 1.3), which employs a validated 4-tissue segmentation method (67). Briefly, participants' T1 image [the four echoes averaged into a root mean square (RMS) image] were first registered to their FLAIR image using FLIRT from FMRIB Software Library version 6.0.1 (68). The FLAIR image was then skull stripped, corrected for inhomogeneities using a previously published local histogram normalization (69), and then non-linearly aligned to a standard atlas (67). WMHs were estimated in standard space using Bayesian probability based on histogram fitting and prior probability maps. Voxels labeled as WMHs based on these maps exceeded 3.5 SDs above the mean WM signal intensity. WMH volumes were calculated in participants' native FLAIR space after back-transformation and reported in cubic millimeters.

### Statistical Analyses

Statistical analyses were performed using SPSS (IBM, Chicago, IL, USA, version 28), with results considered statistically significant at *P* < 0.05. Two main linear regression models were performed. The predictor variable in each model was total ePVS count. The dependent variable in the first model was MoCA score and the dependent variable in the second model was whole brain WMH volume. In the case of significant omnibus results, *post-hoc* comparisons were conducted to assess relationships between ePVS in specific ROIs and MoCA scores or WMH volume. Age, sex, years of education and estimated total intracranial volume were included as covariates in all models. Estimated total intracranial volume (eTIV) was computed using FreeSurfer as described elsewhere (70).

Three additional analyses were run to control for potential confounders in the relationship between ePVS and MoCA scores. In the first follow-up model, we added other cSVD neuroimaging measures as additional covariates (lacune counts and cerebral microbleed counts). In a second follow-up model, we added available self-reported cSVD risk factors as covariates [body mass index (BMI), hypertension, and diabetes]. Finally, due to a correlation between ePVS counts and whole brain WMH volume, *post-hoc* regression models were conducted to determine if the relationships between ePVS in our ROIs and MoCA scores remained significant after controlling for whole brain WMH volume.

All predictors and dependent variables were tested for the assumption of normality using the Shapiro-Wilk test. Collinearity between predictors in all models was explored using the variance inflation factor (VIF), with a value of 5 implemented as a threshold value (71). For the generation of scatterplot figures, predictor and dependent variable scores were z-scored within our participant sample to aid identification of potential outliers.

## Results

### Data Characteristics

High intra-rater reliability was achieved for ePVS (ICC = 0.9) on a subset of 20 randomly selected participants. MoCA scores were skewed (W statistic = 0.914; *P* ≤ 0.001) and therefore log-transformed. The distribution of whole brain WMH volume was also skewed, as is typical (W statistic = 0.542; *P* ≤ 0.001), and thus log-transformed. Variance inflation factor for all predictors was <2 and tolerance was >0.5 in all analyses. Error residuals from all ePVS analyses were normally distributed indicating that the assumption of normality was met.

### Relationship Between ePVS and MoCA

**Figure 2:**
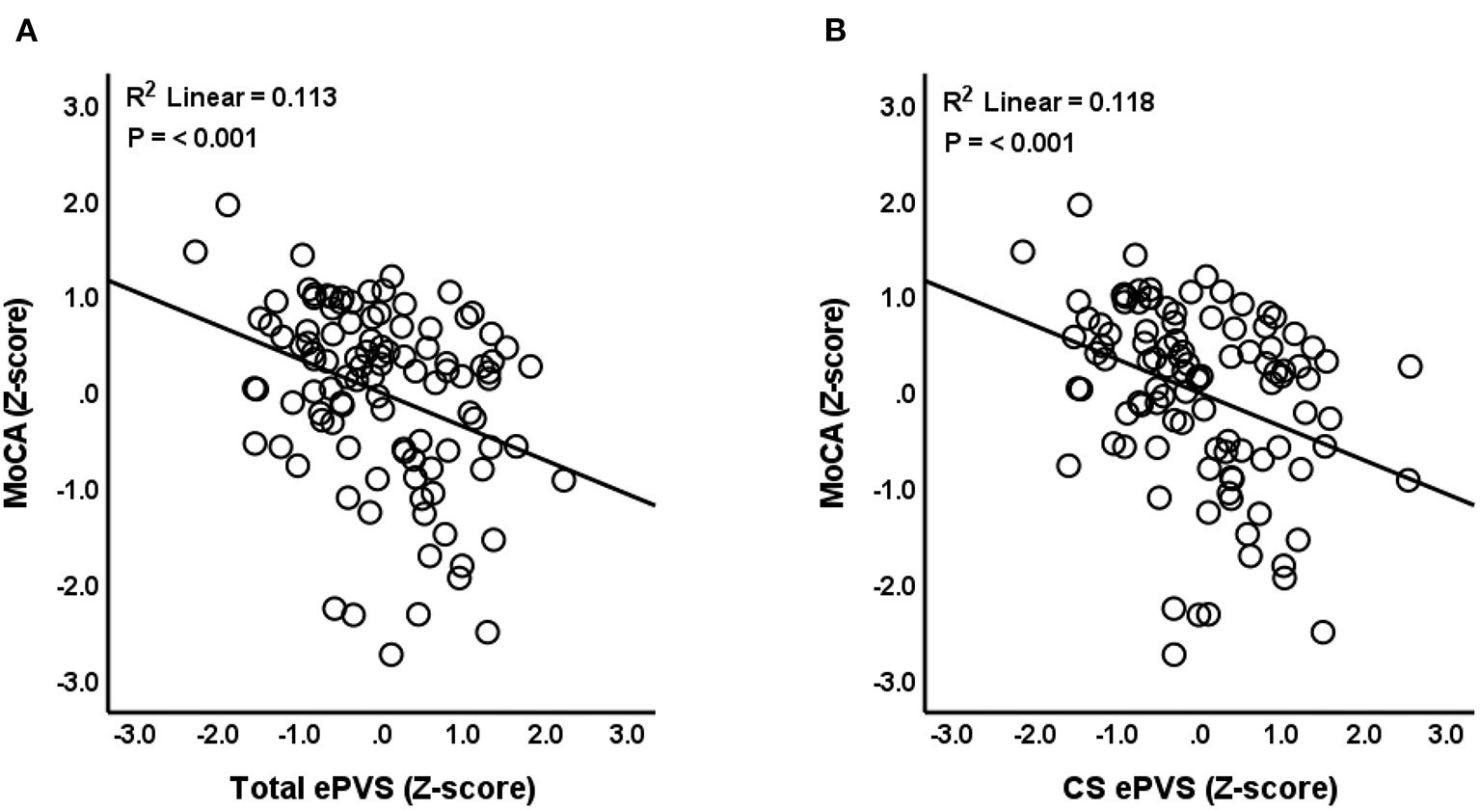
Relationship between ePVS and MoCA scores. The scatterplot shows total ePVS values **(A)** and centrum semiovale (CS) values **(B)** plotted against MoCA scores. Standardized (Z-scores) are presented to identify potential outliers.

Results indicated that total ePVS counts were negatively associated with MoCA scores (*N* = 105, β = −0.352, *P* ≤ 0.001, SE = 0.099, 95% CI = −0.549 to −0.156, VIF = 1.239) after controlling for age, gender, eTIV, and years of education (Figure 2A). *Post-hoc* comparisons were conducted to assess relationships between ePVS in specific ROIs and MoCA scores. Results indicated that number of ePVS in the centrum semiovale was negatively associated with MoCA scores (*N* = 105, β = −0.351, *P* ≤ 0.001, SE = 0.097, 95% CI = −0.543 to −0.160, VIF = 1.179) after controlling for age, gender, eTIV, and years of education (Figure 2B). ePVS in the basal ganglia was marginally associated with MoCA scores (*P* = 0.07), while hippocampus (*P* = 0.561), and midbrain (*P* = 0.447) were not associated with MoCA score.

Next, we assessed the impact of controlling for additional covariates on our observed relationships between ePVS counts and MoCA scores. First, lacunes and cerebral microbleeds, known cSVD neuroimaging markers, were added as continuous covariates. The relationship between total ePVS and MoCA (*P* = 0.002) as well as the relationship between centrum semiovale ePVS and MoCA (*P* ≤ 0.001) remained significant. ePVS in the basal ganglia (*P* = 0.205), hippocampus (*P* = 0.579), and midbrain (*P* = 0.629) were still not associated with MoCA score.

Second, we investigated participant-reported risk factors of cSVD as covariates in the ePVS-MoCA model. BMI was treated as a continuous variable and hypertension and diabetes were treated as dichotomous variables. Once again, the relationship between total ePVS and MoCA (*P* = 0.002), as well as the relationship between centrum semiovale ePVS and MoCA (*P* = 0.001), remained significant. ePVS in the basal ganglia (*P* = 0.145), hippocampus (*P* = 0.629), and midbrain (*P* = 0.542) were not associated with MoCA score.

Neither the addition of other neuroimaging markers of cSVD as covariates in our models, nor the addition of cSVD risk factors, affected the significance of our results. Therefore, we summarize statistical values (Table 2) and present figures (Figure 2) from the original linear regression model which includes age, gender, years of education, and eTIV as covariates.

**Table 2:** Linear regression analyses: relationship between ePVS and MoCA scores or WMH Volume.

**Effect**	**β**	***R*** **^2^**	***P*** **-Value**	**SE**	**95% CI**	
**Model 1: dependent variable—MoCA (N =105)**						
Total ePVS	−0.352	0.113	<0.001^**^	0.099	−0.549	−0.156
CS ePVS	−0.351	0.118	<0.001^**^	0.097	−0.543	−0.160
BG ePVS	−0.186	0.033	0.070	0.102	−0.388	0.016
HPC ePVS	−0.056	0.003	0.561	0.096	−0.246	0.134
MB ePVS	−0.073	0.006	0.447	0.096	−0.264	0.117
**Model 2: dependent variable—WMH volume (N = 104)**						
Total ePVS	0.245	0.069	0.010^*^	0.093	0.060	0.430
CS ePVS	0.206	0.053	0.027^*^	0.092	0.023	0.388
BG ePVS	0.324	0.118	<0.001^**^	0.089	0.148	0.500
HPC ePVS	0.016	<0.001	0.860	0.088	−0.159	0.190
MB ePVS	−0.138	0.025	0.117	0.087	−0.312	0.035

*MoCA, Montreal Cognitive Assessment; ePVS, enlarged perivascular space; CS, centrum semiovale; BG, basal ganglia; HPC, hippocampus; MB, midbrain; WMH, white matter hyperintensity*.

*Both Model 1 and Model 2 adjusted for age, gender, years of education, and eTIV*.

* *P ≤ 0.05*.

** *P ≤ 0.01*.

### Relationship Between ePVS and WMH Volume

**Figure 3:**
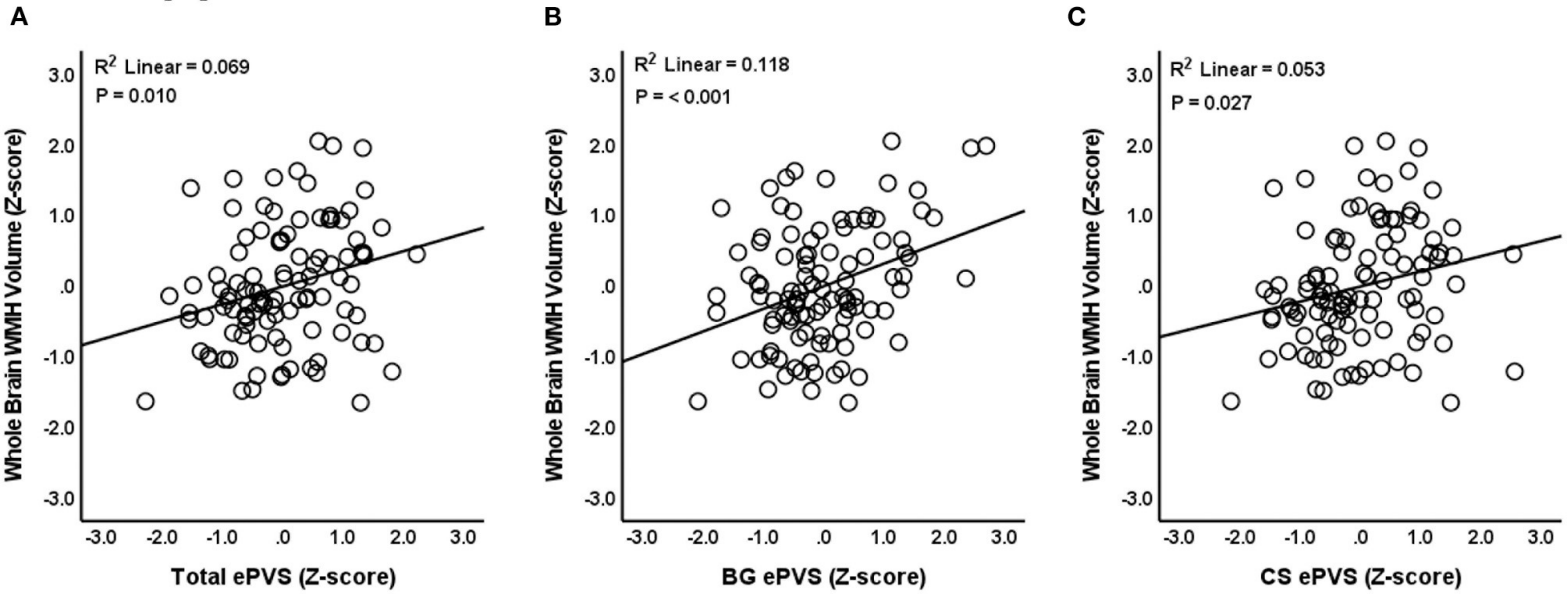
Relationship between ePVS and whole brain WMH volume. The scatterplot shows total ePVS values **(A)**, basal ganglia (BG) values **(B)**, and centrum semiovale (CS) values **(C)** plotted against whole brain WMH volume. Standardized (Z-scores) are presented to identify potential outliers.

Linear regression models were used to evaluate the relationship between ePVS and WMH volume. Results indicated that total ePVS count was positively associated with whole brain WMH volume (*N* = 104, β = 0.245, *P* = 0.010, SE = 0.093, 95% CI = 0.060–0.430, VIF =1.239) after controlling for age, sex, eTIV, and years of education (Figure 3A). *Post-hoc* comparisons indicated that basal ganglia ePVS were positively associated with whole brain WMH volume (*N* = 104, β = 0.324, *P* ≤ 0.001, SE = 0.089, 95% CI = 0.148–0.500, VIF = 1.191) (Figure 3B). In addition, centrum semiovale ePVS were positively associated with whole-brain WMH volume (*N* = 104, β = 0.206, *P* = 0.027, SE = 0.092, 95% CI = 0.023–0.388, VIF = 1.179) (Figure 3C). ePVS in the midbrain (*P* = 0.117) and hippocampus (*P* = 0.860) were not significantly related to WMH volume. Linear regression statistical values for the relationships between WMH volume and ePVS by ROI are reported in Table 2.

### Relationship Between ePVS and MoCA After Controlling for WMH Volume

Consistent with our hypotheses, our results thus far indicate a relationship between ePVS and both MoCA and WMH volume. In order to determine if our hypothesized relationship between ePVS and MoCA is independent of WMH volume, a final set of regression models were conducted between ePVS and MoCA scores after controlling for whole brain WMH volume. Standard covariates of age, gender, eTIV, and education were also included. The relationship between total ePVS and MoCA (*N* = 104, β = −0.358, *P* ≤ 0.001, SE = 0.104, 95% CI = −0.564 to −0.153, VIF = 1.326) as well as the relationship between centrum semiovale ePVS and MoCA (*N* = 104, β = −0.354, *P* ≤ 0.001, SE = 0.100, 95% CI = −0.552 to −0.155, VIF = 1.240) remained significant after controlling for total WMH volume. ePVS in the basal ganglia (*P* = 0.094), hippocampus (*P* = 0.572), and midbrain (*P* = 0.386) were still not associated with MoCA scores. Statistical values for these relationships across all ROIs are reported in Table 3.

**Table 3:** Linear regression analyses: relationship between ePVS and MoCA scores controlling for whole brain WMH volume.

**Effect**	**β**	***R*** **^2^**	***P*** **-Value**	**SE**	**95% CI**	
**Model 3: depdent variable—MoCA (N = 104)**						
Total ePVS	−0.358	0.107	<0.001^**^	0.104	−0.564	−0.153
CS ePVS	−0.354	0.112	<0.001^**^	0.100	−0.552	−0.155
BG ePVS	−0.185	0.029	0.094	0.109	−0.402	0.032
HPC ePVS	−0.055	0.003	0.572	0.097	−0.247	0.137
MB ePVS	−0.085	0.008	0.386	0.098	−0.280	0.109

*MoCA, Montreal Cognitive Assessment; ePVS, enlarged perivascular space; CS, centrum semiovale; BG, basal ganglia; HPC, hippocampus; MB, midbrain; WMH, white matter hyperintensity*.

*Model 3 adjusted for age, gender, years of education, eTIV, and whole brain WMH volume*.

** *P ≤ 0.01*.

## Discussion

We explored cross-sectional relationships between enlarged perivascular spaces (ePVS) and both scores on the Montreal Cognitive Assessment (MoCA) and white matter hyperintensity (WMH) volume. Our results show that a greater ePVS burden in community-dwelling older adults is associated with lower scores on the MoCA, after controlling for age, gender, eTIV and years of education. Additional models controlling for other relevant cSVD variables did not change our results. Further, greater ePVS burden was also associated with higher WMH volume. Our results add to growing evidence that ePVS are clinically significant and may represent an early marker of vascular cognitive dysfunction.

Our results indicate that total ePVS count, assessed within brain regions exhibiting relatively high ePVS burden, was negatively associated with scores on the MoCA in a cohort of community-dwelling older adults. Our results are among the first to our knowledge to demonstrate a negative association between ePVS burden and MoCA scores. Previous studies exploring the association between ePVS with scores on another global cognitive screening tool, the Mini-Mental State Exam (MMSE), have yielded mixed results, as described in the Introduction. The reasons for these mixed results remain unclear but may include lower sensitivity of the MMSE for early cognitive dysfunction compared to the MoCA (43, 72–74). In the present study we were unable to assess the relationship between ePVS and the MMSE because only a subset of participants completed the MMSE and the range of scores was narrow (range = 25–30). Future studies should directly compare the strength of associations between ePVS and both MoCA and MMSE scores.

Our follow-up ROI analyses showed that global cognitive function measured by the MoCA appears to be particularly sensitive to ePVS burden in the centrum semiovale. The relationship between ePVS in the basal ganglia and MoCA approached significance (*P* = 0.07), while neither ePVS in the hippocampus nor the midbrain were associated with MoCA scores. The centrum semiovale is a large area of white matter located above the lateral ventricles and includes projection, association, and commissural tracts. It includes ascending and descending connections between neocortical regions and subcortical regions including thalamus and basal ganglia. As such, the centrum semiovale includes key portions of the fronto-striatal-thalamic pathway, a circuit known to contribute to executive functions (75, 76). Furthermore, previous studies have demonstrated that the integrity of centrum semiovale white matter plays a role in cognition throughout the lifespan (77–80). The present results add to this literature by demonstrating that ePVS burden in the centrum semiovale may have a more prominent effect on global cognitive function assessed by the MoCA than ePVS in other brain regions we investigated.

Next, to anchor our results firmly within the existing ePVS literature, we assessed the cross-sectional relationships between ePVS burden and WMH volume. Our results indicated that total ePVS were positively associated with whole brain WMH volume after controlling for age, gender, eTIV and years of education. These results are in-line with previous reports in cognitively normal participants (81–83), participants with cerebral small vessel disease (14, 84–86), and cognitively impaired participants (22, 23, 33).

Our follow-up ROI analyses showed that ePVS in the centrum semiovale and basal ganglia were positively related to whole-brain WMH volume. This is consistent with previous studies that demonstrated relationships between WMHs and ePVS in the centrum semiovale (82, 86) and between WMHs and ePVS in the basal ganglia (14, 22, 34, 81, 82, 84, 86). Despite tendency for ePVS and WMH volume to co-occur, our *post-hoc* analyses showed that the relationships we observed between ePVS and MoCA remained significant after controlling for WMH volume. While intriguing, this result stemmed from a *post-hoc*, exploratory analysis and will require future longitudinal studies in order to allow interpretation.

Overall, our results suggest that ePVS burden in the centrum semiovale was associated with both general cognitive function and WMH volume whereas ePVS in the basal ganglia was associated with WMH volume. It is possible that ePVS in the centrum semiovale and the basal ganglia may differ in etiology or that this finding may be more related to differential functions of the centrum semiovale and basal ganglia in regards to MoCA performance. Centrum semiovale ePVS have been linked to cerebral amyloid angiopathy and β-amyloid deposition (22, 87–89). In contrast, ePVS in the basal ganglia have been more closely associated with hypertensive arteriopathy (22, 87). The differences between ePVS in the centrum semiovale and basal ganglia should be further explored to understand the independent and synergistic contributions to cognitive dysfunction and WMH burden.

Strengths of our study generally relate to both clinical usefulness and clinical meaningfulness. Clinical usefulness is supported by our use of a validated method for visual rating of ePVS (47, 48) focusing on a single representative slice in key ROIs shown to have the highest ePVS burden. We demonstrated high intra-rater reliability using this method. In addition, the counting procedure does not require advanced neuroimaging analysis skills that may be less typically employed in clinical environments. Similarly, the MoCA enables a brief, sensitive assessment of global cognitive function that is routinely performed in clinical settings (42, 43, 73, 90). Additional strengths include treating ePVS as a continuous variable in our analyses, which more accurately reflects the biologically continuous nature of ePVS burden than categorical rating scales. Finally, our study utilized a moderately large study sample size including a wide age range of older adults.

### Limitations

This study has limitations that highlight the need for additional follow-up studies. First, our cross-sectional study cannot determine if ePVS predict cognitive dysfunction and white matter damage. Future research using longitudinal imaging and clinical data collection is needed to identify if ePVS are baseline predictors of longitudinal cognitive decline and WMH change. While longitudinal studies are the gold-standard for investigating the predictive capacity of ePVS, results from cross-sectional studies such as the present one may prove useful in optimizing the selection of relevant variables for use in future longitudinal ePVS studies. It should also be noted that our cohort included primarily highly educated, White participants. Our findings will need to be replicated in more diverse cohorts. Future studies should also explore the associations between ePVS and MoCA scores in a more clinically heterogeneous set of participants, including those with dementia.

Finally, future longitudinal studies should consider mechanistic contributions to ePVS development. For example, longitudinal studies considering the potential contributions of biofluid markers of AD, atrophy, and inflammatory processes to subsequent ePVS development could prove informative (12, 14, 17, 18, 86, 91–93). In particular, the inflammatory processes of arterial stiffening and blood-brain-barrier breakdown are both established markers of cSVD and should be explored as predictors of ePVS development or as modifiers of ePVS effects on global cognition (10, 17, 24, 94–96). A better understanding of the potential mechanisms of perivascular space enlargement could point to early intervention targets intended to slow or prevent cognitive dysfunction.

### Conclusion

Our results indicate that ePVS burden in older adults is negatively associated with performance on the MoCA, a standardized clinical measure of global cognitive function. Further, ePVS burden was positively associated with WMH volume, an established marker of cSVD. Our results are consistent with a view that ePVS are clinically significant and motivate future longitudinal studies exploring the accuracy of ePVS in predicting subsequent cognitive decline and cSVD progression.

## Data Availability Statement

The raw data supporting the conclusions of this article will be made available by the authors, without undue reservation.

## Ethics Statement

The studies involving human participants were reviewed and approved by Institutional Review Board of the University of Kentucky. The patients/participants provided their written informed consent to participate in this study.

## Author Contributions

TL: conceptualization, data collection, data curation, methodology, formal analysis, writing (original draft), and writing (review and editing). VZ and CB: data collection, data curation, methodology, and writing (review and editing). DW and GJ: data collection, data curation, and writing (review and editing). FR: conceptualization, data collection, methodology, and writing (review and editing). BG: conceptualization, data curation, methodology, formal analysis, writing (review and editing), supervision, project administration, and funding acquisition. All authors contributed to the article and approved the submitted version.

## Funding

This work was supported by the National Institutes of Health (grant numbers NIA R01 AG055449, NIA R01 AG068055, NINDS RF1 NS122028, NIA P30 AG072946, NINDS UH3 NS100606, NINDS UF1 NS125488, NIGMS S10 OD023573, and NIH Training Grant T32 AG 05746105). This work was also supported by an award from the American Heart Association (TL).

## Author Disclaimer

The content is solely the responsibility of the authors and does not necessarily represent the official views of these granting agencies.

## Conflict of Interest

The authors declare that the research was conducted in the absence of any commercial or financial relationships that could be construed as a potential conflict of interest.

## Publisher's Note

All claims expressed in this article are solely those of the authors and do not necessarily represent those of their affiliated organizations, or those of the publisher, the editors and the reviewers. Any product that may be evaluated in this article, or claim that may be made by its manufacturer, is not guaranteed or endorsed by the publisher.
